# Tension Pneumopericardium from a Gastrojejunal-Pericardial Fistula Causing Tamponade: A Case Report

**DOI:** 10.5811/cpcem.63616

**Published:** 2026-08-31

**Authors:** Catherine Moscinski, Alyssa Altheimer, Lachlan Driver, Nikita Maryanov, Cassidy Cunningham

**Affiliations:** Warren Alpert Medical School of Brown University, Department of Emergency Medicine, Providence, Rhode Island

**Keywords:** pneumopericardium, cardiac tamponade, gastrojejunal ulcer, pericardiocentesis, case report

## Abstract

**Introduction:**

Cardiac tamponade typically develops from the accumulation of blood and other fluids, although it can occur from pneumopericardium.

**Case Report:**

A 62-year-old man presented to the emergency department with chest pain and was found to have a pneumopericardium on computed tomography. He developed signs of obstructive shock with hypotension refractory to intravenous fluids and vasopressors. The patient stabilized with bedside pericardiocentesis and pericardial drain placement.

**Conclusion:**

Gastrointestinal-pericardial fistulae are a rare cause of pneumopericardium. Emergency physicians typically rely on cardiac point-of-care ultrasound to evaluate for tamponade and guide pericardiocentesis. However, air artifacts interfere with images, complicating diagnosis and procedures.

## INTRODUCTION

Pneumopericardium, defined as the presence of air within the pericardial sac, is a rare condition in adults that carries significant morbidity and mortality. While it can be self-limiting, it may progress to tension pneumopericardium, resulting in cardiac tamponade, obstructive shock, and death if not promptly recognized.^1^,^2^ Historical mortality rates approach 57%, and tension physiology confers a 56% mortality even with intervention.^1^ Common etiologies include trauma, positive pressure ventilation, iatrogenic causes (particularly atrial fibrillation ablation and cardiothoracic surgery), and, rarely, gastrointestinal-pericardial fistulae.^1^–^3^

Gastrointestinal-pericardial fistulas are exceptionally rare causes of pneumopericardium; only 65 cases are reported in the modern literature, mostly secondary to prior esophageal or gastric surgery.^4^,^5^ Intestinal-pericardial fistulae involving the jejunum are documented only in isolated case reports, making jejunal-pericardial fistula among the rarest etiologies of pneumopericardium.^4^–^6^

Emergency physicians frequently rely on cardiac point-of-care ultrasound (POCUS) for rapid evaluation of hypotension and chest pain, which may be the presenting signs of tension pneumopericardium. However, intrapericardial air poses significant diagnostic challenges. Air is highly hyperechoic and reflects ultrasound waves, creating characteristic “dirty shadowing,” comet-tail artifacts, and loss of visualization of deeper cardiac structures.^7^ This acoustic impedance can obscure the myocardium and pericardial layers, impeding assessment of tamponade physiology and potentially delaying diagnosis.^8^ The classic sonographic finding is the “air gap” sign—bright spots moving along the pericardial layer during diastole with comet-tail artifacts that disappear during systole.^7^ When POCUS is limited by intrapericardial air, computed tomography remains the most sensitive modality for detecting pneumopericardium and delineating fistulous tracts.^4^,^9^

We present a case of pneumopericardium presenting as chest pain and hemodynamic instability, ultimately attributed to a jejunal-pericardial fistula. This case highlights the diagnostic challenges posed by intrapericardial air on cardiac POCUS and underscores the importance of maintaining a broad differential diagnosis for pneumopericardium in the emergency department (ED). This case also underscores the critical importance of maintaining proficiency in landmark-based procedural skills, such as pericardiocentesis, when ultrasound guidance is unavailable.

## CASE REPORT

A 62-year-old male presented to the ED with pleuritic chest pain and dyspnea. The patient’s past medical history included atrial fibrillation, a recent pulmonary embolism managed with apixaban, and an incomplete first through fourth cervical spinal cord injury resulting in quadriplegia. Additional history included obesity status post Roux-en-Y gastric surgery, hypertension, gastroesophageal reflux disease, and diabetes mellitus. Initial blood pressure was 85/59 mm Hg, with tachycardia at 110 beats/min (bpm); subsequent vital signs included blood pressure 56/48 mm Hg and tachycardia at 124 bpm. The patient presented awake, alert, and oriented, although ill-appearing, pale, and diaphoretic. No crepitus or pain to palpation was noted to his chest wall or abdomen. Heart sounds were distant, although no obvious Hamman sign was auscultated, and no jugular venous distention was noted. Lung sounds were clear to auscultation bilaterally. His neurological examination was pertinent for incomplete paraplegia with decreased motor function from his chest to his feet with intact sensation, unchanged from his baseline. Lower extremity examination demonstrated bilateral pitting edema; peripheral pulses were 2+ bilaterally.

Initial resuscitation included intravenous fluids (IVF), followed by norepinephrine vasopressor support at 5 mcg/min with subsequent improvement in his blood pressure. A computed tomography (CT) angiography aortic dissection protocol revealed moderate pneumopericardium and a small pericardial effusion without definite findings to suggest cardiac tamponade (Image 1). A POCUS and formal transthoracic echocardiogram were completed with limited views due to his pneumopericardium. Cardiac windows were intermittently lost with reverberation or dirty shadowing consistent with intrapericardial air, limiting assessment for right ventricle diastolic collapse. The ED clinical team consulted cardiac surgery and thoracic surgery services, both of which recommended conservative management. The patient, however, continued to decompensate, requiring additional IVF and continued norepinephrine for vasopressor support. Interventional cardiology performed an emergent bedside pericardiocentesis as tamponade physiology was assumed, and a pericardial drain was placed to gravity (Image 2).

A CT esophagogram was later completed without radiographic evidence of esophageal perforation. The patient had persistent pneumopericardium and developed recurrent hypotension and tachycardia, concerning for recurrent obstructive shock despite interventions. In addition, he developed agonal breathing and was intubated for airway protection. Bedside aspiration from the pericardial drain removed a significant amount of air from the pericardium, with improvement in his blood pressure. The pericardial drain was connected to a low-suction drainage system. The pericardial fluid was sent for culture, which grew mixed aerobic flora, and he was initiated on vancomycin and piperacillin-tazobactam. Bronchoscopy did not reveal evidence of large-airway injury or a fistula. An elevated pericardial fluid amylase level was noted, concerning for a gastrointestinal source of his pneumopericardium. Repeat CT of the patient’s chest, abdomen, and pelvis was notable for the appearance of a marginal ulcer at the gastrojejunal anastomosis fistulizing through the diaphragm and into the pericardium, creating a jejunal-pericardial fistula.

*CPC-EM Capsule*

What do we already know about this clinical entity?

*Pneumopericardium—the accumulation of air within the pericardial sac—has a mortality rate up to 57% and can progress to tension physiology and tamponade.*

What makes this presentation of disease reportable?

*Pneumopericardium presented as chest pain and hemodynamic instability, ultimately attributed to a jejunal-pericardial fistula, an exceedingly rare etiology.*

What is the major learning point?

*Intrapericardial air can render cardiac ultrasound nondiagnostic. Emergency physicians must maintain proficiency in landmark-based pericardiocentesis.*

How might this improve emergency medicine practice?

*This case reinforces the need to recognize ultrasound limits from intrapericardial air and to maintain competency in blind pericardiocentesis for obstructive shock.*

During admission, the patient underwent an upper gastrointestinal (UGI) endoscopy, which revealed a large ulcer with an 18-mm defect just distal to the anastomosis with visualization of pericardial tissue (Image 3). The surgical team placed an endoscopic vacuum therapy device (Endovac) with a nasogastric tube and surgical sponge attached a wound vacuum over the fistula. Interval UGI endoscopies revealed improvement in the fistula size. Cardiac surgery created a pericardial window with pericardial and pleural drain placement via left anterior thoracotomy. Pericardial fluid samples were obtained and were positive for 1+ yeast (*Candida lambica*) and mold. Antimicrobial coverage was expanded to include micafungin and liposomal amphotericin B. Long-term management included interval Endovac procedures to facilitate fistula closure and completion of a six-week course of antimicrobials.

## DISCUSSION

This case highlights tension pneumopericardium as an uncommon but life-threatening cause of obstructive shock in the ED. The key challenges included recognition of tamponade despite limited echocardiographic windows and identification of an underlying source capable of ongoing air entry into the pericardial space.

Cardiac tamponade typically develops from the accumulation of blood and other fluids. However, there is growing evidence that it can also occur from pneumopericardium, similarly impairing diastolic filling and reducing cardiac output. Although rare, it is relatively more frequent in trauma patients and neonates who require positive pressure ventilation. It can also occur from other causes, including infections leading to fistula formation in contiguous organs, esophageal-pericardial fistulas resulting from caustic ingestions, bronchial carcinoma leading to erosion into the pericardium, ulceration of a pulsion diverticulum, development of gastric-pericardial fistulas from perforated gastric ulcers and eroding gastric carcinomas, and from sepsis, where the pericardium was seeded with gas-producing bacteria.^1^

Management of the pneumopericardium depends on the patient’s clinical presentation but is typically conservative, including serial examinations and monitoring, analgesics, oxygen therapy, and treatment of the underlying condition.^10^ However, patients have a high risk of clinical decompensation, and approximately one in four patients require transfer to the intensive care unit for close monitoring and further interventions.^11^,^12^ In the setting of clinical decompensation, pericardiocentesis is the first-line intervention for immediate decompression and hemodynamic stabilization.^10^ As with our patient, it was critical to recognize the progressive development of obstructive shock and advocate for life saving interventions.

Emergency physicians frequently rely on cardiac POCUS for rapid evaluation of hypotension and chest pain and to assist with the pericardiocentesis. However, intrapericardial air poses significant diagnostic and intraprocedural challenges by creating substantial artifacts and impairing visualization of deeper cardiac structures.^7^ Specifically, intrapericardial air creates significant acoustic impedance, producing reverberation and dirty shadowing that can intermittently eliminate cardiac windows and obscure visualization of right ventricular collapse. When POCUS assessment is limited by air artifact, the absence of classic findings should not be used to exclude clinically significant tamponade physiology. In this setting, CT can be particularly useful for confirming pneumopericardium, evaluating for an associated effusion and identifying potential etiologies, such as fistula formation.

This case highlights the challenges of stabilization and performance of a pericardiocentesis when there is limited echocardiographic visualization. Although uncommon, pneumopericardium warrants inclusion in the differential diagnosis, especially in unstable patients presenting with chest pain and prior gastric surgeries. Patients may require early surgical evaluation and subsequent transfer to a facility with specialized cardiac services. While the pericardial drain was initially attached to a gravity drainage bag with a small amount of pericardial effusion output, pneumopericardium, especially with a fistula as in this case, will likely require the pericardial drain to be attached to low continuous suction to prevent the re-accumulation of air and subsequent recurrence of cardiac tamponade. Given re-accumulation, definitive management often requires source control and urgent surgical evaluation.

## CONCLUSION

Tension pneumopericardium is a rare but life-threatening cause of obstructive shock that can mimic classic fluid tamponade. Intrapericardial air may significantly degrade echocardiographic windows, limiting sonographic assessment and complicating ultrasound-guided pericardiocentesis. In patients with pneumopericardium and worsening hemodynamics—especially those with prior foregut surgery—clinicians should maintain a high index of suspicion for tension physiology and consider prompt decompression based on clinical status, even when ultrasound guidance is not feasible. Definitive management requires identifying and treating the underlying source, and persistent or recurrent pneumopericardium may necessitate continuous drainage and multidisciplinary surgical and endoscopic source control.

## Figures and Tables

**Image 1:**
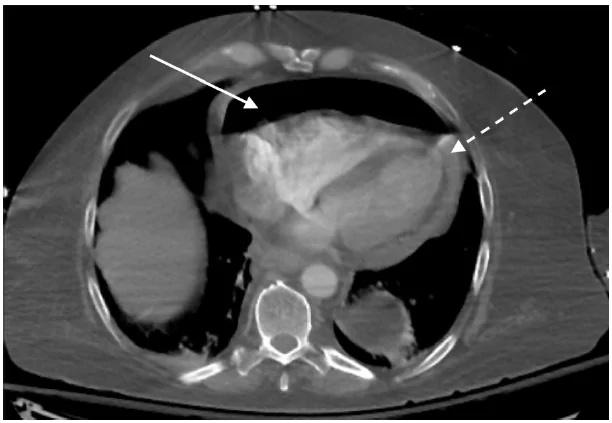
Computed tomographic angiography aortic dissection protocol, in lung window, highlighting a moderate-volume pneumopericardium (solid arrow) with associated small-volume pericardial effusion (dashed arrow).

**Image 2:**
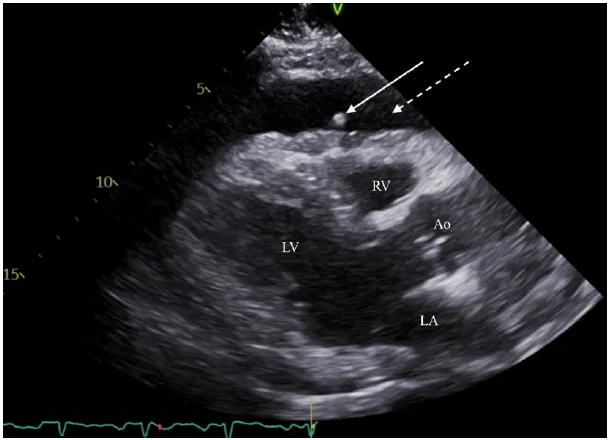
Formal transthoracic echocardiogram in the parasternal long axis following emergent pericardiocentesis with aspiration of air and placement of pericardial drain (solid arrow) with residual pericardial effusion (dashed arrow). Ao, aortic outflow tract; *LA*, left atrium; *LV*, left ventricle; *RV*, right ventricle.

**Image 3:**
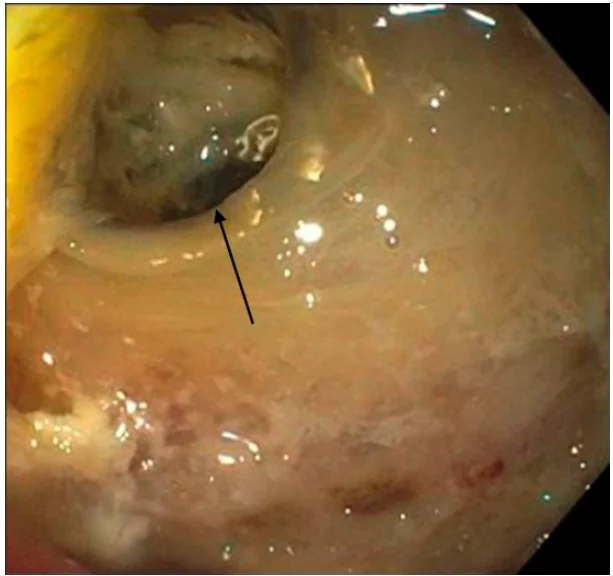
Gastrojejunal-pericardial fistula at the anastomosis of Roux-en-Y gastric surgery (arrow) seen on upper gastrointestinal endoscopy.
